# Metabolic features of cancer cells

**DOI:** 10.1186/s40880-018-0335-7

**Published:** 2018-10-30

**Authors:** Yugang Wang, Yan Xia, Zhimin Lu

**Affiliations:** 1 Brain Tumor Center and Department of Neuro-Oncology, The University of Texas MD Anderson Cancer Center, Houston, TX 77030 USA; 2 Department of Molecular and Cellular Oncology, The University of Texas MD Anderson Cancer Center, Houston, TX 77030 USA; 3 Cancer Biology Program, The University of Texas MD Anderson Cancer Center UT Health Graduate School of Biomedical Sciences, Houston, TX 77030 USA

**Keywords:** Metabolic enzymes, Metabolites, Non-canonical functions, Protein kinase, Lipid metabolism, Gene expression, Anabolism, Catabolism, Redox homeostasis, DNA repair

## Abstract

Cancer cells uniquely reprogram their cellular activities to support their rapid proliferation and migration and to counteract metabolic and genotoxic stress during cancer progression. In this reprograming, cancer cells’ metabolism and other cellular activities are integrated and mutually regulated, and cancer cells modulate metabolic enzymes spatially and temporally so that these enzymes not only have altered metabolic activities but also have modulated subcellular localization and gain non-canonical functions. This review and several others in this issue of *Cancer Communications* discuss these enzymes’ newly acquired functions and the non-canonical functions of some metabolites as features of cancer cell metabolism, which play critical roles in various cellular activities, including gene expression, anabolism, catabolism, redox homeostasis, and DNA repair.

## Introduction

Cancer cells produce energy through the Warburg effect, in which high rates of glycolysis and lactic acid fermentation occur in the cytosol regardless of the oxygen level [1–3]. Promoted by the Warburg effect and other altered metabolic activities, cancer cells have increased anabolism, which includes the synthesis of nucleotides, amino acids, and lipids, alter anti-metabolic stress responses to maintain hemostasis and survival, and reprogram gene expression in a metabolism-dependent way to support their proliferation. Notably, some metabolic enzymes and metabolites in cancer cells have recently been found to have additional functions, which are distinct from their original roles in metabolic reactions, that directly regulate a variety of cellular activities, including metabolism, gene expression, cell cycle progression, apoptosis, autophagy, and exosome secretion [4, 5]. In the current issue of *Cancer Communications*, this review and several others cover these newly identified features of cancer metabolism as well as the regulation of cancer metabolism and other critical cellular functions and highlight the non-canonical functions of metabolic enzymes and metabolites in cancer progression. Particularly, this review also underscore the main themes discussed by other reviews in the same issue.

## Non-canonical functions of metabolic enzymes

Protein kinases regulate intracellular signal transduction pathways and every aspect of cellular activities, whereas metabolic enzymes are traditionally known as the catalyzers of the reactions in cell metabolism [4]. However, we previously demonstrated that metabolic enzymes, such as pyruvate kinase M2 (PKM2), phosphoglycerate kinase 1 (PGK1), and fructokinase-A [also known as ketohexokinase (KHK)-A], can also function as protein kinases. We showed that upon activation of receptor tyrosine kinases, nucleus-translocated PKM2 in a complex with β-catenin phosphorylates histone H3 Thr11 to induce β-catenin-mediated gene expression. This leads to enhanced Warburg effect by upregulating the expression of glycolytic genes and accelerated G1-S transition via cyclin D1 upregulation [6–8]. In mitosis, PKM2 phosphorylates the spindle checkpoint protein BUB3 at Tyr207 to govern kinetochore-spindle attachment and the mitotic checkpoint, thereby promoting accurate chromosome segregation and the proliferation of tumor cells [9]. PKM2 is also involved in cytokinesis. PKM2 interacts with myosin light chain 2 in the contractile ring regions of mitotic cells and phosphorylates it at Tyr118. This phosphorylation promotes the contraction of the actomyosin complex at the cleavage furrow for the completion of cytokinesis [10]. We also demonstrated that PGK1 is a protein kinase to phosphorylate and activates pyruvate dehydrogenase kinase isozyme 1 at Thr338, leading to suppression of mitochondrial pyruvate metabolism. Under energy stress, PGK1 phosphorylates Beclin 1 at Ser30 to promote autophagy. The integrated regulation of glycolysis, mitochondrial metabolism, and autophagy by PGK1 is instrumental to the promotion of tumor cell proliferation and the maintenance of cell homeostasis [11, 12]. In addition to identifying the protein kinase activity of the glycolytic enzymes PKM2 and PGK1, we found that hepatocellular carcinoma cells shut down fructose metabolism by switching their expression of the high-activity KHK-C to that of the low-activity KHK-A isoform. KHK-A in the fructose metabolic pathway phosphorylates and activates phosphoribosyl pyrophosphate synthetase 1 to promote de novo nucleic acid synthesis and hepatocellular carcinoma development [2, 13].

The non-metabolic functions of metabolic enzymes is reviewed by Lu et al. in the current issue of *Cancer Communications*. The metabolic enzyme 6-phosphofructo-2-kinase/fructose-2,6-bisphosphatase 4 (PFKFB4) has both kinase and phosphatase activity. PFKFB4 uses its kinase activity to synthesize fructose 2,6-bisphosphate from fructose-6-phosphate and adenosine triphosphate (ATP) and its phosphatase activity to hydrolyze fructose 2,6-bisphosphate into fructose-6-phosphate and inorganic phosphate [14]. PFKFB4 functions as a protein kinase and phosphorylates steroid receptor coactivator-3 at S857 to activate activating transcription factor 4, leading to the enhanced expression of genes encoding the metabolic enzymes necessary for purine synthesis [14]. We recently demonstrated that the α-ketoglutarate (α-KG) dehydrogenase complex, which is known to catalyze the conversion of α-KG to succinyl-coenzyme A (CoA) in the mitochondria, interacts with the histone acetyltransferase lysine acetyltransferase 2a (KAT2A, also known as GCN5) in the nucleus and locally produces succinyl-CoA. KAT2A uses α-KG dehydrogenase–generated succinyl-CoA for histone succinylation at the Lys79 in the promoter regions of more than 6000 genes, thereby promoting tumor growth [15]. In addition, acetyl-CoA-producing enzymes, such as acetyl-CoA synthetase short chain family member 2 (ACSS2) [16–18], ATP citrate lyase (ACLY) [19, 20], and the pyruvate dehydrogenase complex [21], translocate to the nucleus to regulate gene expression [5]. In response to ionizing radiation-induced DNA damage, fumarase translocates from the cytosol to the nucleus [22], where fumarase binds to the histone variant H2A.Z. Fumarase-produced fumarate at DNA damage regions inhibits α-KG-dependent, lysine-specific demethylase 2B (KDM2B) histone demethylase activity, resulting in increased dimethylation of histone H3K36 and accumulation of the DNA-dependent protein kinase complex for subsequent non-homologous end joining DNA repair and cell survival [23].

Metabolic enzymes can also directly regulate oncogenic signaling. In response to epidermal growth factor receptor (EGFR) activation, the platelet isoform of phosphofructokinase 1 (PFKP), which is a rate-limiting enzyme in glycolysis, is stabilized by the inhibition of ubiquitylation-proteasomal degradation and is acetylated by lysine acetyltransferase 5 at a lysine residue (K395) and translocates to the plasma membrane, where PFKP is phosphorylated at residue Y64 by EGFR [24, 25]. Phosphorylated PFKP binds to a Src homology 2 domain of p85α and recruits p85α to the plasma membrane, resulting in activation of phosphoinositide 3-kinase (PI3K). PI3K-dependent AKT activation promotes glucose transporter 1 (GLUT1) expression and enhances phosphofructokinase 2 (PFK2) phosphorylation and the production of fructose-2,6-bisphosphate, which in turn promotes phosphofructokinase 1 activation and the Warburg effect [25].

Thus, discoveries from our group and others demonstrate that cancer cells alter the subcellular localization of some metabolic enzymes and utilize the non-canonical functions of these enzymes to regulate diverse cellular activities, including the activation of oncogenic signaling, direct regulation of cell metabolism, modulation of gene expression, promotion of cell cycle progression and cytokinesis, and control of DNA repair [26].

## Non-canonical functions of metabolites

Metabolites act as cofactors and the regulators of various enzymes, including those implicated in chromatin modification and gene expression. In this issue of *Cancer Communications*, Wang and Lei summarize the impact that cancer-induced metabolic changes have in altering modifications on histones and DNA [27].

Methyltransferases and demethylases can reversibly regulate the methylation of DNA and histones and subsequent gene expression. S-adenosyl methionine (SAM) is a universal methyl donor and is synthesized from methionine and ATP by methionine adenosyltransferases (MATs) [28]. S-adenosyl homocysteine (SAH), which is a potent inhibitor of all methyltransferases, is formed by the demethylation of SAM. The intracellular SAM/SAH ratio, which is tightly regulated by the metabolism of methionine, threonine, and serine, dynamically regulates methyltransferase activity [29–31]. The removal of histone and DNA methylation by the Jumonji C family members and the ten-eleven translocation (TET) methylcytosine hydroxylases uses a dioxigenation reaction that requires metabolite co-factor α-KG [32], which is produced by glucose and glutamine catabolism. This demethylation can be inhibited by metabolites structurally related to α-KG, including succinate, fumarate, and 2-hydroxyglutarate, the latter of which is a product primarily from the isocitrate dehydrogenase genes *IDH1* and *IDH2* [33, 34]. In addition, high succinate and fumarate levels can be induced by tumor-associated loss-of-function mutations in the succinate dehydrogenase gene *SDH* and fumarase gene *FH* in SDH- and FH-deficient tumors, respectively [31, 35].

Metabolites also serve as substrates or regulators of histone acetyltransferase and histone deacetylase, a pair of enzyme families that acetylate and deacetylate histones, respectively. The metabolic regulation of acetyl-CoA production from acetate, citrate, pyruvate, fatty acid β-oxidation, and the metabolism of amino acid and ketone bodies is instrumental for histone acetyltransferase activity. Accordingly, acetyl-CoA metabolism is tightly regulated both spatially and temporally to elicit responses from the transcription machinery to nutrient availability [5]. In contrast, butyrate and ketone body β-hydroxybutyrate, which is produced by fatty acid hydrolysis, directly inhibit class I and IIa histone deacetylases, and the deacetylases sirtuin 1 and sirtuin 2 are activated by high NAD^+^ levels and inhibited by nicotinamide (NAM), a precursor of NAD [5].

In addition to methylation and acetylation, covalent modifications of DNA and histones, including hydroxylation, crotonylation, β-hydroxybutyrylation, 2-hydroxyisobutyrylation, *O*-GlcNAcylation and polyadenosine diphosphate ribosylation, have been shown to be influenced by metabolic processes [5].

As Xia and Chen elucidate in this issue of *Cancer Communications*, metabolites can be key players in signaling pathways to provide a metabolic advantage to cancer cells, thereby promoting tumor cell proliferation, tumorigenesis, and tumor progression. For instance, ribulose-5-phosphate, an intermediate product of the downstream product of the pentose phosphate pathway, disrupts the active liver kinase B1 (LKB1)-sterile 20-related adapter (STRAD)-mouse protein-25 (MO25) complex and inhibits LKB1-adenosine monophosphate-activated protein kinase (AMPK) signaling. Leucine enhances mammalian target of rapamycin (mTOR) activity, likely by shifting the raptor-mTOR complex from a stable and inactive complex to an unstable and active complex. In addition, acetoacetate, an intermediate of ketogenesis, binds to the BRAF V600E mutant rather than its wild-type counterpart and subsequently promotes BRAF V600E’s binding to its substrate, mitogen-activated protein kinase kinase 1 (MEK1), thereby increasing MEK1/extracellular signal-regulated kinase (ERK)1/2 activity.

Metabolites are also involved in the regulation of metabolic enzymes. Xia and Chen report that the glycolytic intermediate product 3-phosphoglycerate competes with glucose 6-phosphate and inhibits 6-phosphogluconate dehydrogenase in the pentose phosphate pathway. Serine binds to PKM2, promoting the conversion of the PKM2 dimer to a tetramer and decreasing the Km value of PKM2 for its activator, phosphoenolpyruvate.

Thus, metabolites function as cofactors and regulators of metabolic and non-metabolic enzymes and regulate critical cellular activities in both cellular metabolism and other cellular functions.

## Lipid metabolism in cancer

De novo lipid synthesis and uptake, which contribute to the intracellular lipid levels of mammalian cells, are upregulated in cancer cells [36]. Lipids are essential components in membrane biosynthesis, function as second messengers to transduce cellular signals, and serve as important energy sources. In the current issue of *Cancer Communications*, Kuo and Ann discussed recent findings about the regulation of lipid metabolism, especially those regarding sterol regulatory element-binding proteins (SREBPs), which are transcriptional factors that are key players in the transcriptional control of genes that regulate lipid uptake, such as the low-density lipoprotein receptor gene *LDLR*, and genes involved in lipid synthesis, such as those encoding ACLY, acetyl-CoA carboxylase (ACC), and fatty acid synthase (FASN) [36]. SREBP activation is repressed by the ER–resident insulin-induced gene 1 (INSIG1) protein, which binds to SREBP cleavage-activating protein (SCAP) and is modulated by sterols to prevent the translocation and activation of SREBP [36]. The mTOR complex 1-S6K1 interaction is crucial for SREBP activation and sustained lipogenesis and hepatosteatosis under conditions of insulin resistance. In cancer cells, many oncogenic signaling molecules, such as p53, phosphatase and tensin homolog (PTEN), PI3K, and KRAS, converge on the PI3KAKT/mTOR pathway and activate SREBP-mediated lipid biosynthesis to meet lipid demands for cell growth [37]. Oncogenic EGFR signaling increases the *N*-glycosylation of SCAP, which facilitates the release of the SCAP-SREBP1/2 complex from INSIG1, and induces the SREBP1/2-dependent expression of enzymes required for lipogenesis and the expression of low-density lipoprotein receptor for cholesterol uptake [36].

Under the state of fasting or starvation, SREBP1 and lipogenesis are inhibited. AMPK is a highly conserved sensor of cellular energy status. It can directly phosphorylate and suppress SREBP1c gene expression and SREBP-dependent hepatosteatosis [37]. Kuo and Ann describe that under energy stress, fatty acid oxidation, which comprises a cyclical series of catabolic reactions to shorten fatty acids, produces the reduced form of nicotinamide adenine dinucleotide (NADH), 1,5-dihydro-flavin adenine dinucleotide (FADH2), nicotinamide adenine dinucleotide phosphate (NADPH), and ATP. NADH and FADH2 enter the electron transport chain to produce ATP in the mitochondria. NADPH, as a reducing agent, counteracts the increased ROS in cancer cells that result from metabolic stress and hypoxia [38]. Carnitine palmitoyltransferase 1, a rate-limiting enzyme in fatty acid oxidation, conjugates fatty acids and translocates them to the mitochondria to facilitate cancer metabolic reprograming. Lipid droplets are an important source of fatty acid. Understanding the mechanism underlying the lipolysis of lipid droplets in cancer will provide insight into the dynamic regulation of lipid metabolism during cancer growth and progression.

## Redox homeostasis in cancer

Cancer cells have high levels of oxidative stress [39, 40]. This oxidative stress is exerted by accumulated ROS, which is induced by hypoxia, metabolic defects, and endoplasmic reticulum stress [41]. Cancer cells elevate the levels of ROS scavengers for survival in response to the increased ROS levels [41]. Nuclear factor erythroid 2-related factor 2 (Nrf2), a master transcriptional regulator of enzymes, is activated in response to oxidative and electrophilic stress and plays an essential role in cellular redox homeostasis [42]. Elevated Nrf2 activity has been detected in many types of human cancer [42–46]. In the nucleus, Nrf2 regulates the expression of genes, including those encoding NADPH-generating enzymes [47] and those involved in glutathione biosynthesis, to maintain reduced intracellular levels of glutathione. Glutathione and NADPH play essential roles in redox homeostasis and cell survival [48–50].

In glutathione synthesis, the transsulfuration pathway, which links the methionine cycle to glutathione metabolism, converts homocysteine to cysteine, the limiting reagent in the pathway [51]. Cysteine is also provided by solute carrier family 7 member 11 (SLC7A11; also known as system xc^−^), which is a sodium-independent antiporter of cysteine and glutamate and takes up extracellular cysteine in exchange for intracellular glutamate at a 1:1 ratio. As Koppula et al. review in this issue of *Cancer Communications*, SLC7A-derived glutathione can be used by glutathione peroxidase 4 to detoxify lipid hydroperoxides, thereby preventing ferroptosis [52].

Thus, cancer cells reprogram anti-oxidative responses by upregulating Nrf2 activity, the transsulfuration pathway, and SLC7A11 activity, thereby maintaining redox homeostasis for cell survival.

## Conclusions

Cell metabolism and other cellular activities are integrated and mutually regulated, and metabolic enzymes and metabolites have non-canonical functions in these activities. Cancer cells reprogram cellular activities by specifically regulating the expression of metabolic enzymes (e.g., switching from KHK-C to KHK-A expression in hepatocellular carcinoma cells) and altering the subcellular localization of metabolic enzymes such as PKM2, PGK1, fumarase, α-KG dehydrogenase, and ACSS2. These relocalized metabolic enzymes directly govern critical cellular activities to support tumor development in a manner dependent on their originally defined metabolic or non-metabolic enzymatic activity (e.g., protein kinase activity). In addition, metabolic enzymes, such as PFKP, and metabolites, such as acetoacetate, can act as signaling components to directly mediate oncogenic signaling or directly regulate other enzymes’ activities in a subcellular compartment-dependent manner. Metabolism-dependent modifications of key modulators, such as the *N*-glycosylation-dependent activation of SREBP1, control biogenesis and maintain the redox homeostasis of cancer cells. These newly identified features of cancer cell metabolism will facilitate the identification of specific interventions that disrupt these cancer cell-specific features for more efficient cancer treatment.

## References

[1] Yang W, Lu Z (2015). Pyruvate kinase M2 at a glance. J Cell Sci.

[2] Li X, Qian X, Lu Z (2016). Fructokinase A acts as a protein kinase to promote nucleotide synthesis. Cell Cycle.

[3] Li X, Zheng Y, Lu Z (2016). PGK1 is a new member of the protein kinome. Cell Cycle.

[4] Lu Z, Hunter T (2018). Metabolic kinases moonlighting as protein kinases. Trends Biochem Sci.

[5] Wang HL, Wang YL, Zhang HK, Liu ZH, Lu ZW (2018). Determination of thermal focal length under different depths of focus in asymmetrical flat-flat dynamically stable resonators. Opt Laser Technol.

[6] Yang W, Xia Y, Hawke D, Li X, Liang J, Xing D (2012). PKM2 phosphorylates histone H3 and promotes gene transcription and tumorigenesis. Cell.

[7] Yang W, Zheng Y, Xia Y, Ji H, Chen X, Guo F (2012). ERK1/2-dependent phosphorylation and nuclear translocation of PKM2 promotes the Warburg effect. Nat Cell Biol.

[8] Lu Z (2012). PKM2 functions as a histone kinase. Cell Cycle.

[9] Jiang Y, Li X, Yang W, Hawke DH, Zheng Y, Xia Y (2014). PKM2 regulates chromosome segregation and mitosis progression of tumor cells. Mol Cell.

[10] Jiang Y, Wang Y, Wang T, Hawke DH, Zheng Y, Li X (2014). PKM2 phosphorylates MLC2 and regulates cytokinesis of tumour cells. Nat Commun.

[11] Qian X, Li X, Cai Q, Zhang C, Yu Q, Jiang Y (2017). Phosphoglycerate kinase 1 phosphorylates beclin1 to induce autophagy. Mol Cell.

[12] Li X, Jiang Y, Meisenhelder J, Yang W, Hawke DH, Zheng Y (2016). Mitochondria-translocated PGK1 functions as a protein kinase to coordinate glycolysis and the TCA cycle in tumorigenesis. Mol Cell.

[13] Li X, Qian X, Peng LX, Jiang Y, Hawke DH, Zheng Y (2016). A splicing switch from ketohexokinase-C to ketohexokinase-A drives hepatocellular carcinoma formation. Nat Cell Biol.

[14] Dasgupta S, Rajapakshe K, Zhu B, Nikolai BC, Yi P, Putluri N (2018). Metabolic enzyme PFKFB4 activates transcriptional coactivator SRC-3 to drive breast cancer. Nature.

[15] Wang Y, Guo YR, Liu K, Yin Z, Liu R, Xia Y (2017). KAT2A coupled with the alpha-KGDH complex acts as a histone H3 succinyltransferase. Nature.

[16] Li X, Yu W, Qian X, Xia Y, Zheng Y, Lee JH (2017). Nucleus-translocated ACSS2 promotes gene transcription for lysosomal biogenesis and autophagy. Mol Cell..

[17] Mews P, Donahue G, Drake AM, Luczak V, Abel T, Berger SL (2017). Acetyl-CoA synthetase regulates histone acetylation and hippocampal memory. Nature.

[18] Li X, Qian X, Lu Z (2017). Local histone acetylation by ACSS2 promotes gene transcription for lysosomal biogenesis and autophagy. Autophagy..

[19] Sivanand S, Rhoades S, Jiang Q, Lee JV, Benci J, Zhang J (2017). Nuclear acetyl-CoA production by ACLY promotes homologous recombination. Mol Cell.

[20] Wellen KE, Hatzivassiliou G, Sachdeva UM, Bui TV, Cross JR, Thompson CB (2009). ATP-citrate lyase links cellular metabolism to histone acetylation. Science.

[21] Sutendra G, Kinnaird A, Dromparis P, Paulin R, Stenson TH, Haromy A (2014). A nuclear pyruvate dehydrogenase complex is important for the generation of acetyl-CoA and histone acetylation. Cell.

[22] Yogev O, Yogev O, Singer E, Shaulian E, Goldberg M, Fox TD (2010). Fumarase: a mitochondrial metabolic enzyme and a cytosolic/nuclear component of the DNA damage response. PLoS Biol.

[23] Jiang Y, Qian X, Shen J, Wang Y, Li X, Liu R (2015). Local generation of fumarate promotes DNA repair through inhibition of histone H3 demethylation. Nat Cell Biol.

[24] Lee JH, Liu R, Li J, Zhang C, Wang Y, Cai Q (2017). Stabilization of phosphofructokinase 1 platelet isoform by AKT promotes tumorigenesis. Nat Commun.

[25] Lee JH, Liu R, Li J, Wang Y, Tan L, Li XJ (2018). EGFR-phosphorylated platelet isoform of phosphofructokinase 1 promotes PI3K activation. Mol Cell.

[26] Yang W, Lu Z (2013). Nuclear PKM2 regulates the Warburg effect. Cell Cycle.

[27] Gu SM, Han P, Ye ZP, Perkins LE, Li J, Wang HQ (2018). Climate change favours a destructive agricultural pest in temperate regions: late spring cold matters. J Pest Sci.

[28] Grillo MA, Colombatto S (2008). S-adenosylmethionine and its products. Amino Acids.

[29] Mentch SJ, Mehrmohamadi M, Huang L, Liu X, Gupta D, Mattocks D (2015). Histone methylation dynamics and gene regulation occur through the sensing of one-carbon metabolism. Cell Metab.

[30] Shiraki N, Shiraki Y, Tsuyama T, Obata F, Miura M, Nagae G (2014). Methionine metabolism regulates maintenance and differentiation of human pluripotent stem cells. Cell Metab.

[31] Janke R, Dodson AE, Rine J (2015). Metabolism and epigenetics. Annu Rev Cell Dev Biol.

[32] Shi YG, Tsukada Y (2013). The discovery of histone demethylases. Cold Spring Harb Perspect Biol.

[33] Losman JA, Kaelin WG (2013). What a difference a hydroxyl makes: mutant IDH, (R)-2-hydroxyglutarate, and cancer. Genes Dev.

[34] Salminen A, Kauppinen A, Hiltunen M, Kaarniranta K (2014). Krebs cycle intermediates regulate DNA and histone methylation: epigenetic impact on the aging process. Ageing Res Rev..

[35] Sciacovelli M, Goncalves E, Johnson TI, Zecchini VR, da Costa AS, Gaude E (2016). Fumarate is an epigenetic modifier that elicits epithelial-to-mesenchymal transition. Nature.

[36] Cheng C, Geng F, Cheng X, Guo D (2018). Lipid metabolism reprogramming and its potential targets in cancer. Cancer Commun (Lond)..

[37] Shimano H, Sato R (2017). SREBP-regulated lipid metabolism: convergent physiology—divergent pathophysiology. Nat Rev Endocrinol..

[38] Carracedo A, Cantley LC, Pandolfi PP (2013). Cancer metabolism: fatty acid oxidation in the limelight. Nat Rev Cancer.

[39] Cairns RA, Harris IS, Mak TW (2011). Regulation of cancer cell metabolism. Nat Rev Cancer.

[40] Holmstrom KM, Finkel T (2014). Cellular mechanisms and physiological consequences of redox-dependent signalling. Nat Rev Mol Cell Biol.

[41] Gorrini C, Harris IS, Mak TW (2013). Modulation of oxidative stress as an anticancer strategy. Nat Rev Drug Discov..

[42] Sporn MB, Liby KT (2012). NRF2 and cancer: the good, the bad and the importance of context. Nat Rev Cancer.

[43] Wang XJ, Sun Z, Villeneuve NF, Zhang S, Zhao F, Li Y (2008). Nrf2 enhances resistance of cancer cells to chemotherapeutic drugs, the dark side of Nrf2. Carcinogenesis.

[44] Jiang T, Chen N, Zhao F, Wang XJ, Kong B, Zheng W (2010). High levels of Nrf2 determine chemoresistance in type II endometrial cancer. Can Res.

[45] DeNicola GM, Karreth FA, Humpton TJ, Gopinathan A, Wei C, Frese K (2011). Oncogene-induced Nrf2 transcription promotes ROS detoxification and tumorigenesis. Nature.

[46] Inami Y, Waguri S, Sakamoto A, Kouno T, Nakada K, Hino O (2011). Persistent activation of Nrf2 through p62 in hepatocellular carcinoma cells. J Cell Biol.

[47] Mitsuishi Y, Taguchi K, Kawatani Y, Shibata T, Nukiwa T, Aburatani H (2012). Nrf2 redirects glucose and glutamine into anabolic pathways in metabolic reprogramming. Cancer Cell.

[48] Moscat J, Karin M, Diaz-Meco MT (2016). p62 in cancer: signaling adaptor beyond autophagy. Cell.

[49] Katsuragi Y, Ichimura Y, Komatsu M (2015). p62/SQSTM1 functions as a signaling hub and an autophagy adaptor. FEBS J.

[50] Hayes JD, Dinkova-Kostova AT (2014). The Nrf2 regulatory network provides an interface between redox and intermediary metabolism. Trends Biochem Sci.

[51] Prudova A, Bauman Z, Braun A, Vitvitsky V, Lu SC, Banerjee R (2006). S-adenosylmethionine stabilizes cystathionine beta-synthase and modulates redox capacity. Proc Natl Acad Sci USA.

[52] Koppula P, Zhang Y, Zhuang L, Gan B (2018). Amino acid transporter SLC7A11/xCT at the crossroads of regulating redox homeostasis and nutrient dependency of cancer. Cancer Commun (Lond).

